# Low-Latency Line Tracking Using Event-Based Dynamic Vision Sensors

**DOI:** 10.3389/fnbot.2018.00004

**Published:** 2018-02-19

**Authors:** Lukas Everding, Jörg Conradt

**Affiliations:** ^1^Department of Electrical and Computer Engineering, Neuroscientific Systemtheory, Technical University of Munich, Munich, Germany

**Keywords:** robotic vision, silicon retina, neuromorphic sensors, event-based vision, low-level feature extraction, line detection, line tracking

## Abstract

In order to safely navigate and orient in their local surroundings autonomous systems need to rapidly extract and persistently track visual features from the environment. While there are many algorithms tackling those tasks for traditional frame-based cameras, these have to deal with the fact that conventional cameras sample their environment with a fixed frequency. Most prominently, the same features have to be found in consecutive frames and corresponding features then need to be matched using elaborate techniques as any information between the two frames is lost. We introduce a novel method to detect and track line structures in data streams of event-based silicon retinae [also known as dynamic vision sensors (DVS)]. In contrast to conventional cameras, these biologically inspired sensors generate a quasicontinuous stream of vision information analogous to the information stream created by the ganglion cells in mammal retinae. All pixels of DVS operate asynchronously without a periodic sampling rate and emit a so-called DVS address event as soon as they perceive a luminance change exceeding an adjustable threshold. We use the high temporal resolution achieved by the DVS to track features continuously through time instead of only at fixed points in time. The focus of this work lies on tracking lines in a mostly static environment which is observed by a moving camera, a typical setting in mobile robotics. Since DVS events are mostly generated at object boundaries and edges which in man-made environments often form lines they were chosen as feature to track. Our method is based on detecting planes of DVS address events in x-y-t-space and tracing these planes through time. It is robust against noise and runs in real time on a standard computer, hence it is suitable for low latency robotics. The efficacy and performance are evaluated on real-world data sets which show artificial structures in an office-building using event data for tracking and frame data for ground-truth estimation from a DAVIS240C sensor.

## 1 Introduction

This article introduces an algorithm that is aimed at detecting and tracking visual line features with low latency and without requiring much prior knowledge about the environment. We envision this algorithm to be useful toward enabling high-speed autonomous machines to orient in and interact with their environments, e.g., *via* line-based SLAM (Smith et al., 2006). To tackle these tasks the development of low-latency algorithms is required that find a compressed representation of the observed surroundings based on which we can let the autonomous systems move and let make decisions. Feature detectors and trackers can serve as the first step toward this representation. They allow, e.g., an understanding of which parts of a scene move coherently and, therefore, likely belong to the same physical object and which parts belong to distinct entities.

### 1.1 Dynamic Vision Sensors

We use dynamic vision sensors (DVS, see Lichtsteiner et al., 2008; Posch et al., 2011; Brändli et al., 2014) to gather visual data. In contrast to conventional cameras, DVS do not rely on frames, but generate a quasi continuous flow of vision information. Whereas frame-based cameras read out all pixels periodically, pixels of an event-based sensor operate asynchronously and a pixel will generate an event as soon as it undergoes a change in brightness (ON event for increase, OFF event for decrease, respectively). Their response time typically lies in the order of tens of microseconds. Furthermore, frame-based cameras read out all pixels regardless of what is happening in their field of view. This results in creation of redundant information in parts of the image without change, but will lose all information between two consecutive frames in the areas where changes occur; so there is redundancy and information loss at the same time. A lot of computing power is required to separate the useful parts from the scene from the redundant ones. This separation step is not necessary when using DVS. These sensors emit events only when they perceive a brightness change. This mitigates both the redundancy, since there will be no events, as long as no change is perceived, and the information loss because there is no fixed sampling time. These advantages make DVS especially suited for low-latency and high-speed applications as was already demonstrated by, e.g., Delbruck and Lang (2013) and Conradt et al. (2009).

**Figure 1:**
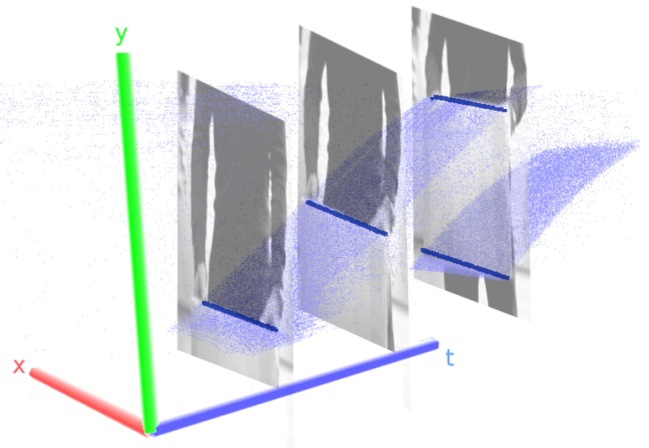
Event traces for a box moved from bottom to top through the field of view of a DVS. Visible are dense manifolds of events corresponding to the two edges of the box. Events originating from the movement of the person holding the box are excluded for the sake of clearer visualization. The frames put in the event stream show snapshots of the situation at the time they were triggered. Box edges are indicated by blue bars for better visibility. Note that one axis corresponds to time!

To illustrate the difference between conventional cameras and DVS further, imagine a wall in front of which a box is moved through a camera’s field of view as an example (cf. Figure 1). A frame-based camera will capture a collection of (likely blurred) snapshots of different positions of the box along its trajectory. They each contain the information about the background and the momentary position of the box, but the information about the position of the box between the frames is lost. In contrast, pixels of a dynamic vision sensor will send events as soon as the box enters and leaves their respective field of view and remain silent at all other times. The event stream contains the position of the box with a time-resolution on the microsecond scale but no redundant information about the static parts of the scene.

The event stream is however still a raw data source from which no useful higher-level information can be trivially gained. In order to arrive at a meaningful interpretation and make the benefits of the DVS accessible it is necessary to cluster the events and assign them to physical origins. This article proposes a step toward this goal and introduces an algorithm for the fast extraction and persistent tracking of lines using dynamic vision sensors.

### 1.2 Line Detection and Tracking

We operate on the assumption that a major part of DVS events belonging to a scene originate from object boundaries because that is where sharp transitions in brightness often occur. While not all boundaries are straight, many are (at least approximately), especially in man-made environments, e.g., for robots moving in indoor scenarios. This makes lines a good feature to track. The goal of our method is to parametrize these lines using the parameters line midpoint $\vec{p}$, line vector $\vec{l}$, and line length *a*. The detection and tracking algorithm leverages the DVS properties in the following way: events are processed independently as soon as they are generated, so we can keep a low latency. The constant influx of events permits to incrementally update our belief about the current state of the environment. Once a line is detected, we can hold onto it as long as it is visible, instead of having to match corresponding lines between subsequent frames. That way we save the computational complexity that an additional interframe matching and tracking step would introduce.

The algorithm requires little prior knowledge about the scene. It is, however, designed for environments which contain many straight edges, but which may also contain arbitrary other objects and non-straight distractors. The lines are assumed to move in a translatory fashion, the method is not designed to detect and track fast spinning lines like the ones produced by fans or rotation of the sensor around the optical axis as may occur when airborne drones perform rolling maneuvers.

ON and OFF events are processed separately. y separating polarities, we gain the benefit of sparser populated x-y-t spaces (one for each polarity). This simplifies line identification as it allows for more generous thresholds, since on average half of the noise and objects have been removed. The lines themselves contain only one polarity and the number of events forming them will therefore not be reduced. Such separate processing of ON and OFF contrast was found to occur in nature and has been studied, e.g., in insect and mammal eyes (Franceschini et al., 1989; Borst and Helmstaedter, 2015) where ON and OFF pathways split in the early processing stages. This concept has been used in multiple bio-inspired robotic experiments for almost thirty years. Franceschini et al. (1992) successfully constructed a robot which autonomously moved while avoiding obstacles based on the visual system of the blow fly. Subsequent robotic experiments extended functionality, while making use of ON/OFF contrast separation. Franceschini (2014) presents an overview, Roubieu et al. (2013) give details on the ON/OFF detection sensor principle. Bagheri et al. (2017) adapted the dragonfly’s motion detector principles to develop a target tracker using frame-based data; Clady et al. (2015) and Vasco et al. (2016) used polarity separation for corner detection in the event-based domain.

The rest of this work is structured as follows: the remainder of Section 1 gives an overview over which line detecting and tracking algorithms have already been developed for classical cameras as well as for event-based sensors, Section 2 will explain the event-based data and the core novelty of this work in depth—the algorithm for line detection and tracking. Experiments and results follow in Section 3. Section 4 concludes the article by indicating how this work can be used and be further developed.

### 1.3 Related Work

There is a variety of algorithms to extract lines from frames, most notably the Hough transform (Duda and Hart, 1972; Matas et al., 2000). In Grompone von Gioi et al. (2012), a line segment detector (called LSD) is proposed that works stably without parameter tuning (see also Section 3 for comparisons). In Section 3, we compare the results of these algorithms with our method. Different methods that use line segments for interframe tracking are described in Neubert et al. (2008), Hirose and Saito (2012), and Zhang and Koch (2013).

In recent years, several trackers for different shapes have been developed for event-based systems. An early example of this can be found in Litzenberger et al. (2006). Based on this, Delbruck and Lang (2013) shows how to construct a robotic goalie with fast reaction time of only 3 ms. Conradt et al. (2009) focuses explicitly on detecting lines from events and describes a pencil balancer. Estimates about the pencil position are performed in Hough space.

In a more recent work, Brändli et al. (2016) describe a line segment detector to detect multiple lines in arbitrary scenes. They use Sobel operators to find the local orientation of events and cluster events with similar angles to form line segments. Events are stored in a circular buffer of fixed size, so that old events are overwritten when new ones arrive and the position and orientation of lines is updated through this process, but do not put their focus on tracking (see Section 3 for a comparison with the here proposed method).

There are also increasing efforts to track other basic geometric shapes in event-based systems: corners have been a focus in multiple works as they generate distinct features that do not suffer from the aperture problem, can be tracked fast and find usage in robotic navigation. Clady et al. (2015) use a corner matching algorithm based on a combination of geometric constraints to detect events caused by corners and reduce the event stream to a corner event stream. Vasco et al. (2016) transfer the well-known Harris corner detector (Harris and Stephens, 1988) to the event domain, while Mueggler et al. (2017) present a rapid corner detection method inspired by FAST (Rosten and Drummond, 2006), which is capable of processing more than one million events per second.

Lagorce et al. (2015) introduces a method to track visual features using different kernels like Gaussians, Gabors, or other hand designed kernels. Tedaldi et al. (2016) uses a hybrid approach combining frames and event stream. It does not require features to be specified beforehand but extracts them using the grayscale frames. The extracted features are subsequently tracked asynchronously using the stream of events. This permits a smooth tracking through time between two frames.

## 2 Materials and Methods

### 2.1 Event-Based Vision Data

Dynamic vision sensors are a novel type of optical sensor whose working principle has been inspired by mammal retinas. The pixels of DVS operate asynchronously and independently from each other. A pixel generates a so called address event as soon as it senses a change in log luminance above a certain threshold rather than measuring actual intensity. An event contains the position on the retina $\vec{x}=\left(x,y\right)$, the time *t* at which it was generated and the polarity *p*, that encodes whether the luminance became higher (ON-event) or lower (OFF-event). The entirety of pixels generates a quasicontinuous stream of events which conveys visual information about the scene. Because these streams carry only information over changes in the environment and no redundant information about static features, DVS generate relatively low data rates. The streams can be represented as point clouds in a three dimensional space with the pixel positions *x* and *y* as well as time *t* as coordinates. We will refer to this space as *x-y-t-space*. There is no notion of frames in DVS data; all event stream images in this work were created by accumulating events for a certain amount of time.

**Figure 2:**
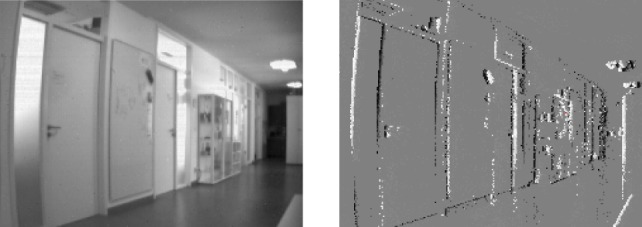
Davis recorded scene: gray scale frame (left), events (right; events have been accumulated for 50 ms; ON events are depicted white, OFF events black, gray areas did not emit any events in the previous 50 ms; camera was rotated clockwise.).

The fact that the sensor uses log luminance gives it a very high dynamic range of about 120 dB (Brändli et al., 2014) and allows it to work reliably in environments with strongly varying illuminations where conventional cameras would experience either over- or underexposure in parts of the image. Figure 2 shows a frame captured with a conventional system and an accumulation of an event stream of the same scene for comparison.

In this work, we use a Davis240C (Brändli et al., 2014) as it is capable of capturing events as well as global shutter gray scale images with the same chip. It has a resolution of 240 × 180 pixels. The algorithm proposed in this article only makes use of the address events, the gray scale images were used to obtain ground truth values to evaluate the performance.

### 2.2 Algorithm

**Figure 3:**
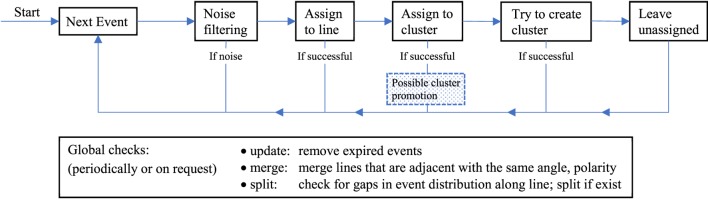
Overview over the algorithm. Top: stream part and bottom: batch part running in background.

The main idea behind the algorithm is to identify planes of events in x-y-t space. On short time scales, straight physical edges move with near constant velocity through the field of view, i.e., acceleration due to physical acceleration or projective transformation can be neglected if the observed time interval is sufficiently small. Therefore, straight edges leave traces of events in x-y-t space that are approximately planar on short time scales. Figure 1 shows event traces of a box that was moved upwards. Note that the duration of this recording would not be considered a short timescale and that the manifolds are slightly curved. Our algorithm aims to identify these manifolds by piecewise approximation with a chain of planes. Each plane is used to infer the movement of a line in the field of view. Note that this approach does not work with spinning lines and will fail to track these as they do not form planes in x-y-t-space. The detection approach is split into two steps: first, we cluster events that were generated within a small time and space window. These clusters are grown by adding events that are close in x-y-t-space. Second, we periodically check if the clusters form planes in x-y-t-space. If they do, we promote the cluster to a line, otherwise we keep them as clusters. Tracking of lines follows by assigning newly incoming events to planes and tracing these planes through time. To avoid that the number of events taken into account in our method is strongly dependent on sensor velocity we use an event buffer with a fixed amount of events at all times and discard old events. Figure 3 shows an overview over the complete processing flow that each incoming event will be subject to. Every event is run through a noise filter, then we check if it belongs to a line and, if so, assign it to this line. Else, we check if a cluster is nearby and if that is the case add the event to the cluster. If there is no cluster, nearby events are collected to form a cluster; if we do not find more events than a threshold *ν*, we leave the event unassigned (*ν* typically lies in the order of 20–30 events). We will explain every step in detail now.

#### 2.2.1 Event Preprocessing

First, events are separated by polarity. After separating events, we apply different noise filters: first, we introduce a refractory period per pixel. After a pixel emitted an event, we will suppress further events from this pixel within a certain time interval, because pixels sometimes generate additional spurious same-polarity events if they have been triggered before and pixels may emit multiple events of the same polarity if the change in brightness was very strong. Experimentally, we identified 1 ms for opposite polarity events and 50 ms for same polarity events to work well. All events that are received during this period with respect to their polarity are discarded. Afterward, an additional filtering step checks for every incoming event if at least 3 same polarity events in a 5 × 5 pixel window around it have been registered. If not, the event is labeled as noise and not processed further.

In the following, we will continue to explain the algorithm from the end of the processing chain, because it is easier for the reader to follow the whole process starting with the way clusters are initially formed, then promoted to lines, and how these lines are finally transferred through time.

#### 2.2.2 Cluster Creation

When an event arrives (and could not be assigned to an existing line or a cluster), we use it as seed to search for a chain of adjoining pixels that recently generated events. First, we search for the youngest event in the ring of the 8 adjacent pixels. If we find no event, we search in the next ring of 16 pixels around the adjacent pixels. If we still find no events, we abort the search. Otherwise, we add the youngest event to our chain and repeat the procedure from the pixel position of the found event. This step is iterated until the chain length crosses a threshold or we do not find any new events.

If we are able to create a chain of events, we cluster the events of the chain, add all events that have been generated by adjacent pixels and store these events as cluster, thereby creating a candidate for a plane.

#### 2.2.3 Cluster Growth

Moving one step to the left in the process flow (Figure 3): when an event could not be assigned to a line, we look for a cluster to attach it to. To that end, we check if there is an event that belongs to an adjacent cluster. If there is, we assign the event to the cluster. If there is more than one cluster found, we merge these clusters and add the event to the new larger cluster.

#### 2.2.4 Cluster Promotion

When a cluster has collected enough events (in the order of 20–40 events), we check if its events form a plane in x-y-t-space. As stated above, the underlying assumption is that the velocity of lines on the retina can be approximated as constant on short time scales. Then, non-spinning straight edges in the real world generate flat planes of events. To check if the candidate cluster’s events form a line, we compute the principal components of the event coordinates (*x, y, t*) where we scale the time coordinate with a constant factor. A value of $1000\frac{\mu \text{s}}{\text{px}}$ was experimentally determined to work well for the robotic platform as well as for handheld movements (in high speed environments this factor may have to adapted):

(1) $$X=\left(\begin{array}{ccc} x_{1} & y_{1} & t_{1}\\ x_{2} & y_{2} & t_{2}\\ \ldots & & \end{array}\right),$$

be the matrix, that contains the coordinates of events belonging to the cluster. Assume, that all coordinates (*x, y, t*) are centered (i.e., $\bar{x}=\sum_{i}\;x_{i}=0$, etc.). It then holds that,

(2) $$\frac{1}{N}X^{T}X=\frac{1}{N}\left(\begin{array}{ccc} \Sigma_{i}\;x_{i}^{2} & \Sigma_{i}\;x_{i}y_{i} & \Sigma_{i}\;x_{i}t_{i}\\ \Sigma_{i}\;x_{i}y_{i} & \Sigma_{i}\;y_{i}^{2} & \Sigma_{i}\;y_{i}t_{i}\\ \Sigma_{i}\;x_{i}t_{i} & \Sigma_{i}\;y_{i}t_{i} & \Sigma_{i}\;t_{i}^{2} \end{array}\right)=U\Delta U^{T},$$

where *i* runs over all events and *N* is the total number of events. For the last step the eigen decomposition was applied. Δ is a diagonal matrix containing the eigenvalues of **X***^T^X*, which are guaranteed to be positive real numbers since **X***^T^X* is real symmetric and positive semidefinite. **U** is a orthogonal matrix containing the respective eigenvectors. As a perfect plane forms a linear 2D subspace in the 3D space, one principal value (eigenvalue) vanishes, when we compute the principal component analysis (PCA) on points lying in the same plane. The corresponding eigenvector stands orthogonally on the plane. Event planes in a real world recording, however, will never be perfect since these planes contain noise through various sources like finite size pixels, electronic jitter, delay during event generation, not perfectly constant velocity of observed objects, events stemming from other sources, etc. For a planar structure one eigenvalue will nevertheless be very small, proportional to the “thickness” of the plane. So, assuming that the eigenvalues in Δ are ordered in ascending order, we examine Δ_11_: if it is greater than a threshold *θ*, we conclude that the cluster does not form a plane and, therefore, was not caused by an observed line. We keep on collecting events and trying to promote it until *n* promotion attempts failed. Then, we drop it (for our experiments, we chose *θ* = 1px^2^ and *n* = 3).

If the smallest eigenvalue is below the threshold we promote the candidate to a real line (or 3D-plane, respectively). The position and orientation of the line at time *t*_present_ is now inferred by intersecting the approximated event plane with the plane *t* = *t*_present_, further called the *present plane*. The parameters to be determined are a vector $\vec{l}$ pointing along the line, a point $\vec{p}$ which is contained in the line and *a* the length of the line. We start finding $\vec{l}$ by using the principal component vector belonging to the smallest eigenvalue as normal vector $\vec{n}\in R^{3}$. The calculation is straightforward and can be done analytically: we use the fact that a vector $\vec{l}$ pointing along the intersection of two planes must lie within both planes and therefore be orthogonal to both plain normals. A possible choice that fulfills this condition is the cross product of event plane normal $\vec{n}$ and present plane normal *e_t_*:

(3) $$\vec{l}=\vec{n}\times {\vec{e}}_{t}={\left(n_{1},n_{2},n_{3}\right)}^{T}\times {\left(0,0,1\right)}^{T}={\left(n_{2},-n_{1},0\right)}^{T}.$$

Having found a vector pointing along the line, the next step is to find a point $\vec{p}$ that is contained by the line. We pick $\vec{p}$, such that it is closest to the events’ center of gravity $\vec{c}=\left(\bar{x},\bar{y},\bar{t}\right)$. $\vec{p}$ can then be found using these two observations: first, the vector $\vec{m}$ pointing from $\vec{c}$ to $\vec{p}$ must be contained in the event plane as $\vec{p}$ and $\vec{c}$ are both contained in this plane, i.e., $\vec{m}$ is perpendicular to the plane normal $\vec{n}$. Second, $\vec{m}$ points along the path of shortest distance from $\vec{c}$ to the line, therefore it has to be perpendicular to $\vec{l}$ as well. This makes $\vec{m}=\vec{n}\times \vec{l}$ a natural choice. We then find $\vec{p}$ as intersection of the line $\vec{c}+\alpha \;\vec{m}$ with the plane *t* = *t*_present_ where we determine *α* using the fact that ${\vec{p}}_{3}=t_{\text{present}}$ and ${\left(\vec{n}\times \vec{l}\right)}_{3}=-n_{1}^{2}-n_{2}^{2}$:

(4) $$\vec{p}=\left(\begin{array}{c} \bar{x}\\ \bar{y}\\ \bar{t} \end{array}\right)-\frac{t_{\text{present}}-\bar{t}}{n_{1}^{2}+n_{2}^{2}}\;\vec{m}.$$

Finally, to find the length *a* of the line we assume that the events are evenly distributed along the whole extension of the line. The length *a* is then proportional to the SD along the line which we already gained from the principal component analysis. We pick the center of the line as origin and use *ξ* as a one dimensional coordinate along the line and a constant distribution function *p*(*ξ*) over the length of the line *a*:

(5) $$p\left(\xi \right)=\left\{\begin{array}{cc} \frac{1}{a},\; & \text{if}-a∕2\leq \xi \leq a∕2\\ 0,\; & \text{otherwise}\\ \; \end{array}\right..$$

Then ⟨*ξ*⟩ = 0 and

(6) $$\left\langle {\left(\xi -\left\langle \xi \right\rangle \right)}^{2}\right\rangle =\left\langle \xi^{2}\right\rangle =\int_{-\infty }^{\infty }\;p\left(\xi \right)\;\xi^{2}\;\text{d}\xi =\int_{-a∕2}^{a∕2}\;\frac{1}{a}\xi^{2}\;\text{d}\xi =\frac{1}{12}a^{2},$$

from which follows that $a=\sqrt{12\;\left\langle \xi^{2}\right\rangle }$.

We arrived at a line parametrization with midpoint $\vec{p}$, direction $\vec{l}$ and length *a*. Note that we chose *t* = *t*_present_ arbitrarily! We can use any other time to predict the line parameters in the (near) future without requiring any new input. The corresponding calculations are as outlined only a few three dimensional vector operations and very fast to perform.

Whenever a cluster is promoted to a line we check if the new line’s position and slope match the ones of a line that was previously deleted. We require the polarities to be identical, the angular distance to be less than 5° and the midpoints distance to the other line to be less than 2px (although this threshold should be adapted depending on the sensor resolution). If there is a deleted line that matches, we assume that we lost track of it and recovered it now. In this case we will assign the new line the ID of the deleted line.

#### 2.2.5 Line Growth

When a new event is received, we check for lines close to the pixel of event generation. We assign the event to the line if it is closer than $\sqrt{\theta }$, with *θ* the threshold for cluster promotion, to the line inferred at the time of event generation. The threshold could however be fixed independently to make the line collect more or less events generated in its surroundings.

#### 2.2.6 Persistent tracking

For larger time spans, the assumption of planarity is violated. This means the principal component analysis breaks down, if events that are too old are used. Therefore, we need to update the inferred planes either periodically or on request, as soon as an accurate estimate is required, by removing events that are older than a certain time or if a line contains many events per length simply by removing the oldest events. After removing these events, orientation of the event plane (and thereby also of the inferred line) will be re-estimated by re-applying PCA and going through all the additional steps described above. Note, furthermore, that this is not an expensive update, since we can store the sum of coordinates for the PCA, and just modify it when adding or removing events from the line. If after an update there remain less than 10 events or the smallest eigenvalue exceeds *θ*, the line will be deleted. We keep deleted lines’ position and orientation in memory in case they are recovered. The following global checks run additionally in the background:

- clusters are also periodically cleaned by removing old events.
- lines are checked for coherence: if lines display gaps in the event distribution, they are split into two lines. Gaps are detected by projecting every event position onto the parametrized line, partitioning the line in bins of stepwidth 2px. If two adjacent bins are empty the line is split at this gap.
- merging of lines: if lines have an angular difference of less than 5° and same polarity, as well as the midpoints’ distances to the respective other line are less than 2px (same values as for recovering deleted lines) and the midpoints’ distances to each other are less than the half sum of the lengths, i.e., the lines are adjacent to each other, they are merged to form just one line.

## 3 Experimental Results

We performed experiments to evaluate the quality of the matching and tracking, as well as quantifying the latency and computational costs and investigated the robustness. For all experiments we used an Intel i7 Core 4,770 K running at 3.5 GHz. The algorithm was implemented without parallelization in C++.

### 3.1 Quality of Matching and Tracking

To evaluate the quality, we recorded data sets with the DAVIS240C, capturing both events and frames (cf. Supplemental Material, frames captured with a rate between 15 and 20 Hz). To our knowledge, there exists no data set with ground truth values for event-based line tracking. So, we obtained ground truth values for lines by applying the well-known Canny edge detector (Canny, 1986) to every frame. Since there is no definite standard to define and extract line from images, we took the Canny filter output and manually removed bent edges based on human judgment, such that only straight edges (i.e., lines) remained. We then applied linear regression to the coordinates of pixels that belong to lines and arrived at line parametrizations which were used as ground truth. Additionally, we manually labeled the lines of all frames so that corresponding lines got the same ID in every frame to allow to check persistence of tracking of our algorithm.

**Figure 4:**
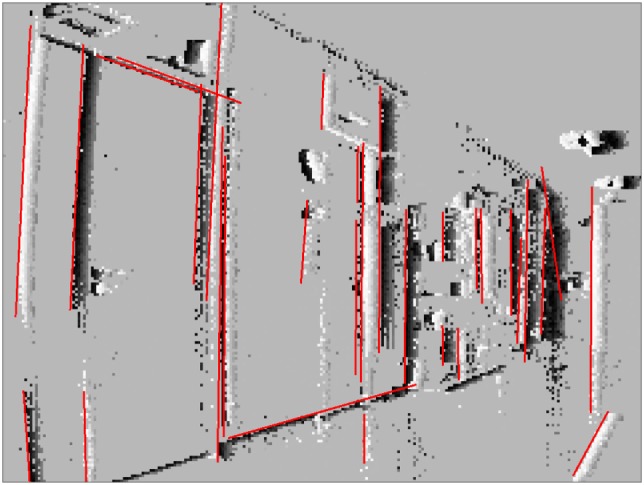
Event stream with current position of detected lines (events accumulated for 50 ms). Camera rotates clockwise, so lines move to the left and older events trailing to the right of lines are still visible.

**Figure 5:**
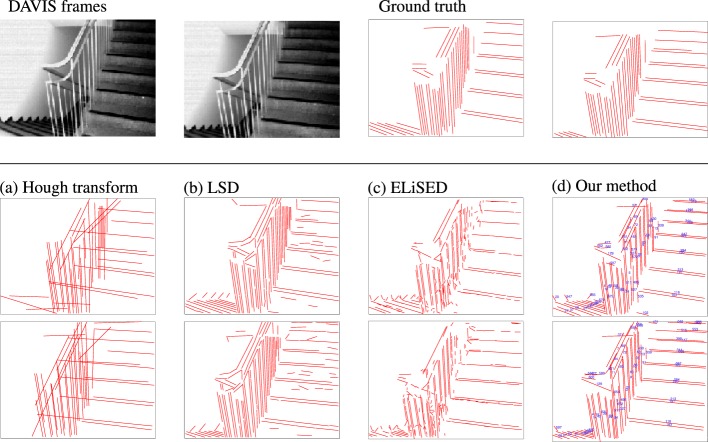
Comparison between different approaches. Top row left-to-right: frame taken from a DAVIS240C recording, frame 200 ms later, ground truth lines for the first frame, ground truth lines for the second frame. Second row left-to-right: (a) Hough transform, (b) LSD, (c) ELiSeD, and (d) our method. Third row: same algorithms as above applied to the second frame. In the images of our method lines were additionally assigned an ID to demonstrate the tracking capabilities (cf. text).

Our method is able to successfully extract lines from event-based vision streams. Figure 4 displays a snapshot of detected lines in an event stream. Figure 5 compares our results to other methods for line detection on two frames with a 200 ms time interval between them using a scene with a staircase which contains many lines. The first row shows the two frames and the corresponding ground truth. The second (resp. third row) show different line detection algorithms applied to the first (resp. second) frame or event stream, depending on algorithm. The leftmost pictures depict the results of the probabilistic Hough transform (Matas et al., 2000) as implemented in OpenCV^1^; the images next to them contain the results of the Line Segment Detection (LSD) (Grompone von Gioi et al., 2012) as provided on the IPOL webpage^2^; followed by ELiSed (Brändli et al., 2016) as implemented in jAER^3^ and finally our method (to arrive at the plot for ELiSeD we took all lines that were created from the time the frame was taken up until 10 ms earlier). The probabilistic Hough transform (a) is able to detect well contrasted lines, but it runs into problems when estimating the right lengths. The detection images look fairly different from the ground truth. Furthermore, the extracted lines look quite different between the frames what would make interframe matching a hard task. In contrast, the line segment detection method (b) yields high-quality results. The extracted line segments reflect the ground truth very well. However, longer lines tend to be broken up into segments and would need to be merge before they can be used for tracking in a next step. ELiSeD (c) tends to yield very small segments and to break up longer lines into smaller pieces. That produces lots of very short-lived segments. Meanwhile, the method proposed here (d) is able to successfully extract longer lines and keep track of them. The numbers on the rightmost panels denote IDs we assigned lines after creation. Most of the IDs of corresponding lines in both panels are identical, i.e., they have been successfully tracked. This tracking did not require an additional step but is gained automatically by applying our algorithm.

**Figure 6:**
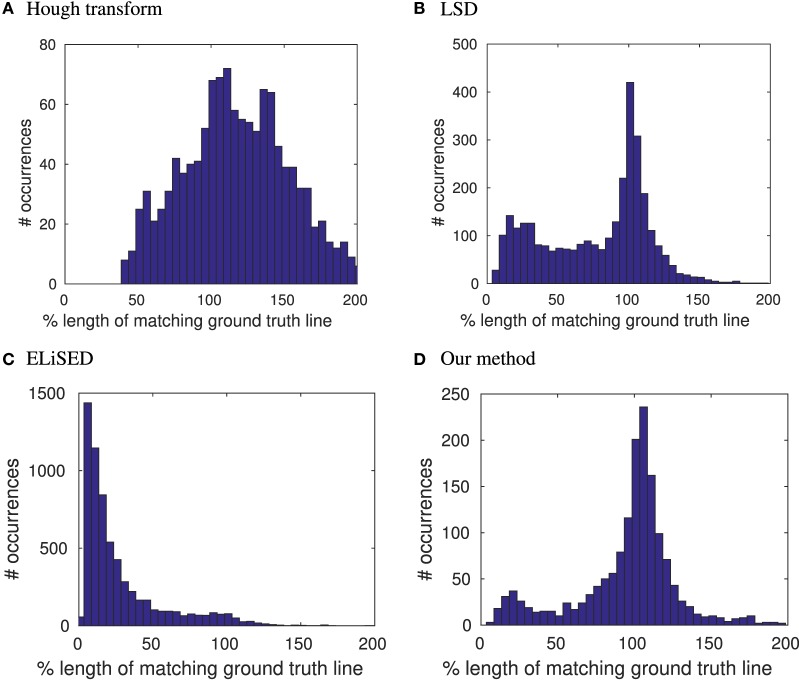
Distributions of length ratios between estimated lines and matching ground truth lines for **(A)** Hough transformation, **(B)** LSD, **(C)** ELiSeD, and **(D)** our method in percentage.

To measure the quality, we compared the estimated lines with the labeled ground-truth lines, where we assumed that an estimated line matches a ground-truth line if their difference of angles was less than 5° and the perpendicular distance from the midpoint of the estimated line to the ground-truth line was smaller than 1.5 px. We then obtained difference of angles and lengths for the matched lines: angles of lines are known to be a robust feature to estimate when detecting lines. This holds also for our method where the average absolute angular error over all matched lines was approximately 0.6°, the median absolute angular error approximately 0.4° (on the same data set Hough had mean/median angular error 0.7°/0.4°, LSD had mean/median angular error 0.9°/0.4° and ELiSeD had mean/median angular error of 1.5°/1.1°). In contrast, line length is a rather unstable feature to extract. Using our algorithm, we are able to extract lines with a high precision of length. Figure 6 shows a distribution of the relative lengths of the estimated lines to the ground truth lines on 52 labeled frames of the staircase data set. For every match of an estimated line with a ground truth line, we calculated the ratio $\frac{\text{length }(\text{estimated line})}{\text{length }(\text{ground truth line})}$ and created a histogram over all matched lines. The distribution for our method peaks comparatively sharply around 100% which means, that the majority of our line estimates are correct in length. LSD also exhibits a peak around 100% but has a tail toward zero, which is caused by estimated lines broken up in segments. ELiSeD produces many small segments leading to a distribution where most matches only cover a small part of the ground truth line and the Hough transformation has big problems estimating lengths, over- and underestimating very often.

### 3.2 Persistence of Tracking

**Figure 7:**
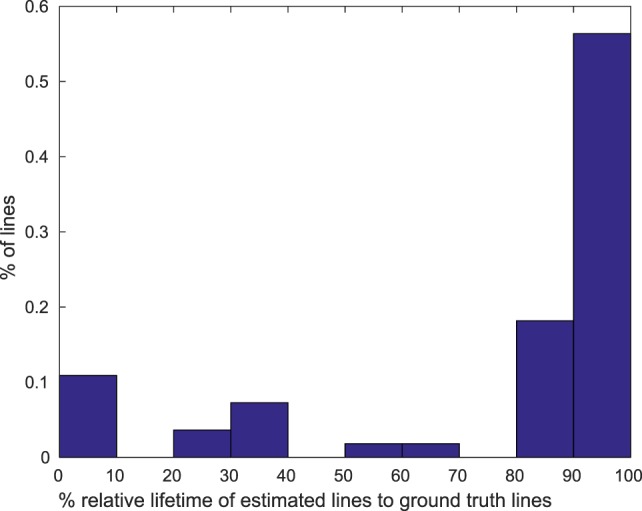
Histogram over ratios of lifetimes of estimated lines to life time of ground truth lines in percentage.

The other aspect we aimed toward besides lines detection was tracking, i.e., we should be able to identify every line over the entire time that it is visible with the same ID. In Figure 5, we attached ID numbers to the lines detected with our algorithm. Most lines that correspond to the same physical lines have the same ID in both images (and at every point in time between the two frames as well (not visible)). To get a better impression on how persistent the tracking is we recommend having a look at the Supplemental Videos. In those you can see that most detected lines keep the same ID over the whole length of the recordings. To evaluate the persistence quantitatively, we took the difference of the first time a ground-truth line was matched by a specific ID with the last it was matched by this ID and compared it with the duration the ground-truth line was in the field of view. Figure 7 shows the distribution of relative lifetimes. More than half of the lines in our experiments are tracked throughout their whole life time; that means they are found very fast after they enter the field of view and subsequently tracked until they leave it. For a smaller fraction of lines it took longer to identify and track them, so that they are not tracked over their whole visibility, while an even smaller fraction of lines was not found quickly or lost during tracking.

**Figure 8:**
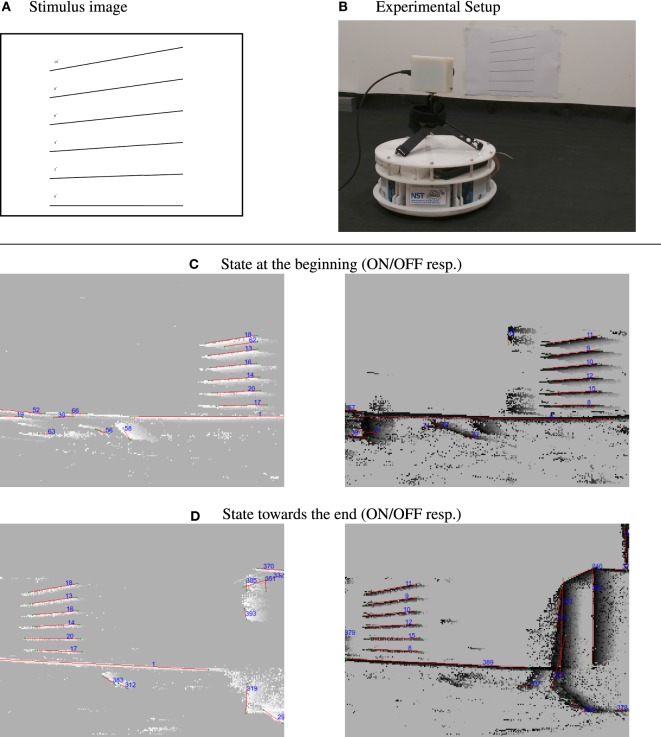
Dependence of line tracking on line orientation. Top row: **(A)** stimulus used: lines with increasing degree versus the camera movement in steps of 2° and **(B)** camera setup on robotic platform, robot was moving to the right during recording. Second/third row: **(C)** tracking results at the beginning of the recording for ON/Off events. **(D)** Tracking results toward the end of the recording. Comparing the IDs, that the lines were signed in the images, it can be seen that they were successfully tracked despite being close to parallel to the movement direction of the sensor (lines become visible due to microvibrations of the robot).

There are certain limitations when working with the DVS. The amount of events that lines generate depends on their angle to the movement direction of the camera. Lines perfectly aligned with the sensor movement direction are invisible to the DVS because only the leading edge presents a luminance change. Therefore, these lines can not be tracked. If a line is being tracked but becomes aligned with the movement after a movement direction change of the sensor it will become invisible, too, and track will be lost. To overcome this, one could include an inertial measurement unit (IMU) and estimate the invisible line’s position using acceleration information. However, it turns out that the problem of invisible lines is in practice not severe. Vibrations of the sensor, which can stem for example from a robot’s motors or natural tremor in case of handheld DVS, cause the sensor to perform movements in the orthogonal direction of the line which makes them visible to the sensor. We performed an experiment to examine the dependence of the line detection on the angle for which we printed lines with known inclination from 0° to 10° in steps of 2° and recorded the scene using a self-built robotic platform with a mounted DVS that drove in parallel to the stimulus. Figure 8 shows the stimulus and the robot we used. Furthermore, it shows the detected lines at the beginning of recording as well as roughly 4 s later at the end of the recording. All lines could be detected, even the line with 0° was tracked because the microvibrations of the robot caused small perpendicular movements which rendered it visible. In fact, these movements generate ON as well as OFF events, leading to a redundant double tracking of the lines due to the fact that we split handling of ON and OFF events. This redundancy can potentially be used to recover from the loss of tracking in one polarity domain. The feasibility of this was not evaluated and will be subject to further investigation.

**Figure 9:**
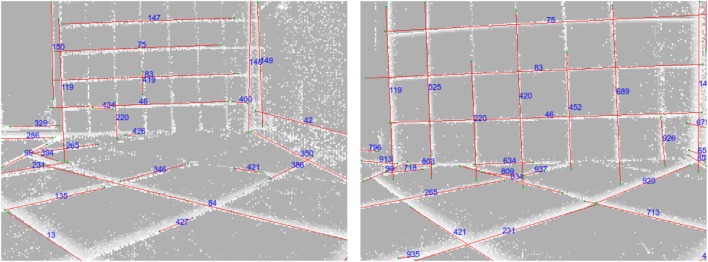
Line tracking results for a robot driving over small irregularities caused by a tiled floor. Comparing line IDs shows that lines were tracked even when crossing seams (only ON events).

In the second experiment, we drove with the robot over the seams of a tiled floor. These irregularities in the surface caused small abrupt movements of the sensor. Figure 9 shows tracked lines in such a setting. It contains two snapshots made by a robot driving toward a tiled wall and crossing seams in the floor on its way. Our method is capable of dealing with small irregularities and small amplitude shaking as can be caused by crossing seams. Sudden changes in movements (like large sudden displacements caused by stronger shaking) can, however, not be dealt with and result in the loss of track of lines. The underlying reason for this is that sudden changes will cause a kink in the event trace in x-y-t-space. These kinks can not be modeled well with the chain of planes and our method will fail. As soon as the lines’ movement is smooth again, they will typically be regained fast, but with a new ID.

As third experiment, we attached a sensor to a radio controlled model car to evaluate behavior at high velocity (~12 km/s). We recorded two different settings and evaluated the results by visual inspection; the recordings are provided as Supplementary Material.

**Figure 10:**
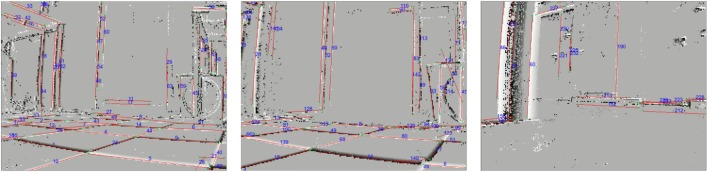
Snapshots from a sensor mounted on an RC car driving over even floor through a door (time increases from left to right, also see Supplementary Material for the recording).

In the first experiment, the car started on a checkerboard pattern floor and drove through a door toward another door. Because the floor was smooth, we observed no major disturbances (especially no abrupt changes in motion) and detection and tracking yielded good results. Figure 10 presents snapshots from this experiment.

In the second scene, the car drove over uneven floor in a narrow hallway with a comparatively high noise level due to irregular illumination patterns and textures on wall and floor. In this recording detection of lines again yielded good results; tracking, however, was more challenging. While some lines (especially those perpendicular to the car vibrations) could be tracked well, others were often lost. This can be explained by the same reason for which we lost track of lines while driving over seams: the car experienced abrupt changes of motion, which lead to kinks in the event plane causing our tracking method to fail. Due to the dynamic nature of the recordings we recommend viewing the video of the experiments provided in Supplementary Material.

### 3.3 Latency and Computing Costs

**Figure 11:**
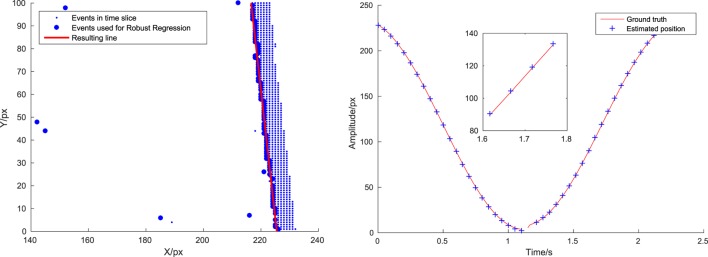
Left: detail from method of ground truth estimation. Right: true line position (red line) and position estimates (blue crosses) at time of availability. Inlay zooms to region between 1.6 and 1.8 s. Position estimate overestimates true position by a small margin.

This section presents an experiment to evaluate the latency. The independent operation of DVS pixels generates a quasicontinuous stream of events. Due to this sensor property the events have already a low delay from illumination change in the scene to reception of the event in a processing device. Each event can be handled individually and is used to update our belief about the current state of the world immediately after arrival. We measured the time it takes to process an incoming event and call the update function that reestimates the line position using a scene with a swinging pendulum. This gives us a single line traversing the display with predictable translatory speed. To measure the error we obtained ground truth values for the line position in the following way: first, we discarded the OFF events and binned the ON events in slices of 50 ms. We then found the leading edge by picking for every pixel row of the sensor the event that was furthest in movement direction. We used robust linear fitting as built-in in MATLAB to fit a line and reject outliers and inspected each fitted line visually. Figure 11 (left) shows one fitted line graphically.

We compared the position at which our algorithm estimated the line with two different approaches: (1) retrieving the position of the line by interpolating the line movement linearly from the last calculated position of the line until the time of position request and (2) calling the update routine and refitting the line before returning the position estimate. For the case of event processing without line update the average required time was approximately 0.7 *μ*s with an estimation error of 0.50 pixels. When we do an explicit line position re-estimation using PCA and vector recalculation as described in Section 2.2, the average time required was approximately 7 *μ*s and the average estimation error was 0.48 pixels. So, the latency between arrival of new information (incoming event) and new belief (updated line state) lies in the order of a few microseconds. Note that we normally do not upgrade after each event! The reason why explicit updating does not reduce the error significantly for the pendulum lies in the fact that the line is very well visible and moves very smoothly, so the interpolation of its movement is already quite accurate. Figure 11 (right) shows the estimated line position at certain arbitrarily chosen checkpoints versus the true line position for the case with line update for a fixed value of y. The red line shows the position of the line, while the blue crosses indicates the position estimated by our algorithm at the time after the computation (i.e., at the time of the event + the time required to estimate the position). The computing time is sufficiently low to make the estimation error introduced by the delay negligible.

**Figure 12:**
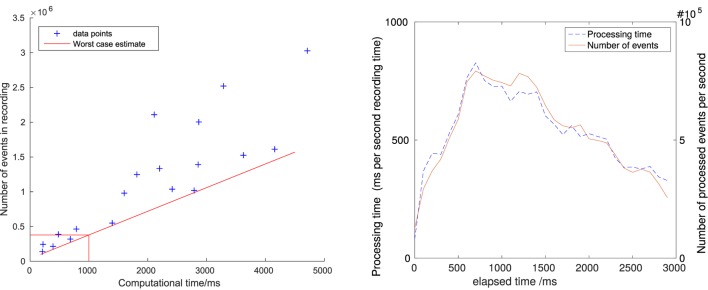
Left: dependence between computing time and number of events for a number of different recordings. Right: processed events per second and required computing time for line tracking in the staircase scene.

In addition to latency, the overall computation load is another important quantity for judging the usefulness of an algorithm. Figure 12 shows the dependence between number of events and required computing time for a number of different recordings for a variety of scenes. The red line gives an estimate for the worst case performance, suggesting that the algorithm can handle at least 400k events per second in the current implementation. On the right hand side, the computing load during one recording with a large number of lines (the staircase scene) is shown. The recording was partitioned in slices of 100 ms; the computation time and number of events in every slice was then measured and plotted in the figure. The graph shows an approximately linear relation between number of events and time required to process them.

## 4 Discussion

We have introduced an algorithm for the fast detection and persistent tracking of translating lines for a biologically inspired class of optical sensors, dynamic vision sensors (DVS). The nature of DVS data allows to solve both tasks, detection and tracking, in a combined approach in which we first cluster events and check for linearity and then continuously grow detected lines by adding events. Additional benefits we can derive from the use of DVS are on the one hand low-latency responses, because DVS pixels emit address events asynchronously as soon as they perceive an illumination change. We made use of this property by processing each event individually and showed in experiment 3.3 that it is possible to determine a line’s position within a few microseconds at arbitrarily chosen points in time with a subpixel accuracy.

On the other hand, our method can potentially be applied in environments with vastly varying lighting conditions, because DVS are insensitive to absolute illumination. This makes it suitable to be employed on robots that work in environments where lighting conditions are not well predictable or unknown beforehand. The efficacy of our method was demonstrated and the results compared to other methods for line detection in frames and in address event streams. Our method performed as well as classical algorithms applied to frames; note however, that classical algorithms are fundamentally constrained to frames and therefore cannot make use of the advantages of the neuronal sensor of low-latency and robustness to lighting variations.

The algorithm is resilient against small displacements and vibrations; vibrations are actually helpful by making more visual features of a scene accessible and allow for their detection and tracking as shown in Section 2. The vibrations make features appear in the OFF and ON stream which enables us to do redundant tracking and potential recovery from lost track in one of the streams which would improve the overall robustness. Sudden larger movements will, however, lead to failing of the tracking as observed lines will no longer form planes in x-y-t space, but exhibit kinks which violates our assumptions. For the same reason of non-planarity, the algorithm is not suited for detecting spinning lines. Other methods need to be developed to handle these. Finally, we will note that the algorithm relies on some parameters which have to fixed beforehand. Some are chip and optics dependent and have to be adapted to the hardware used (like the threshold for line creation and event-to-line assignment and the event number thresholds, e.g., for creating/deleting clusters/lines). Others are environment related, e.g., the number of simultaneously considered events is largely environment dependent, and need to be adapted to the nature of the expected observed scenes; the same holds for the conversion factor from time to pixel for the PCA. The fixed value we used yielded good results for the velocity ranges of our robotic platforms (up to 12 km/s) and handheld cameras. If changing to a high speed environment this factor will likely have to be revisited.

There are a couple of different directions in which to continue. By linking lines that move coherently, reconstructing outlines of objects with straight edges like doors and boxes (or objects whose outlines can be piecewise linearly approximated) can become possible and the algorithm can be developed toward object tracking. Furthermore, matching lines across streams from different DVS could allow for depth estimates. A different direction of advancement would be to extend the promotion mechanism of cluster by introducing PCA kernels for different shapes, e.g., circles. This would allow active systems to not only orient themselves on lines but provide them with more and more distinct features to allow a more robust position estimate and safer navigation. Objects that are not straight, however, behave more complicated under projective transformations, and require more complex parametrizations. There is a variety of options to use our algorithm as a basis for methods that can be used in robotics.

## Author Contributions

LE: designed and implemented algorithm, evaluated performance, and wrote manuscript JC: guided method development and contributed to manuscript and figures.

## Conflict of Interest Statement

The authors declare that the research was conducted in the absence of any commercial or financial relationships that could be construed as a potential conflict of interest.

## Footnotes

^1^https://github.com/itseez/opencv.

^2^http://www.ipol.im/pub/art/2012/gjmr-lsd/.

^3^https://sourceforge.net/projects/jaer/.

## Supplementary Material

Video S1 Staircase.mp4.

Video S2 Corridor.mp4.

Video S3 Office.mp4.

Video S4 RCcar_even_surface.mp4.

Video S5 RCcar_uneven_surface.mp4.
